# Development of 5-Fluorouracil/pH-Responsive Adjuvant-Embedded Extracellular Vesicles for Targeting α_v_β_3_ Integrin Receptors in Tumors

**DOI:** 10.3390/pharmaceutics16050599

**Published:** 2024-04-29

**Authors:** Jiseung Kim, Eunsol Lee, Eun Seong Lee

**Affiliations:** 1 Department of Biotechnology, The Catholic University of Korea, 43 Jibong-ro, Bucheon-si 1462, Gyeonggi-do, Republic of Korea; wltmd98@naver.com (J.K.); eunsollee13@gmail.com (E.L.); 2 Department of Biomedical-Chemical Engineering, The Catholic University of Korea, 43 Jibong-ro, Bucheon-si 1462, Gyeonggi-do, Republic of Korea

**Keywords:** functional extracellular vesicles, pH-responsive adjuvant, 5-fluorouracil, α_v_β_3_ integrin receptors, tumor treatment

## Abstract

To selectively target and treat murine melanoma B16BL6 tumors expressing α_v_β_3_ integrin receptors, we engineered tumor-specific functional extracellular vesicles (EVs) tailored for the targeted delivery of antitumor drugs. This objective was achieved through the incorporation of a pH-responsive adjuvant, cyclic arginine-glycine-aspartic acid peptide (cRGD, serving as a tumor-targeting ligand), and 5-fluorouracil (5-FU, employed as a model antitumor drug). The pH-responsive adjuvant, essential for modulating drug release, was synthesized by chemically conjugating 3-(diethylamino)propylamine (DEAP) to deoxycholic acid (DOCA, a lipophilic substance capable of integrating into EVs’ membranes), denoted as DEAP-DOCA. The DOCA, preactivated using *N*-(2-aminoethyl)maleimide (AEM), was chemically coupled with the thiol group of the cRGD-DOCA through the thiol–maleimide click reaction, resulting in the formation of cRGD-DOCA. Subsequently, DEAP-DOCA, cRGD-DOCA, and 5-FU were efficiently incorporated into EVs using a sonication method. The resulting tumor-targeting EVs, expressing cRGD ligands, demonstrated enhanced in vitro/in vivo cellular uptake specifically for B16BL6 tumors expressing α_v_β_3_ integrin receptors. The ionization characteristics of the DEAP in DEAP-DOCA induced destabilization of the EVs membrane at pH 6.5 through protonation of the DEAP substance, thereby expediting 5-FU release. Consequently, an improvement in the in vivo antitumor efficacy was observed for B16BL6 tumors. Based on these comprehensive in vitro/in vivo findings, we anticipate that this EV system holds substantial promise as an exceptionally effective platform for antitumor therapeutic delivery.

## 1. Introduction

The design of functional drug carriers has garnered significant attention as a crucial strategy to surmount the limitations of chemotherapeutics and the challenges associated with selective targeting [1,2,3,4]. It is recognized that the reactivity of nano-sized particles toward tumor cells is influenced by the chemical structure, shape, charge, and other physical characteristics inherent to these particles [1,4,5,6]. Consequently, precisely engineered nano-sized particles can exhibit unique physical, chemical, and biological properties, rendering them versatile for the attainment of diverse physiological objectives [7,8]. Recent research has been prolific in exploring drug carriers based on extracellular vesicles (EVs) to develop an advanced drug delivery system adhering to essential standards, including biocompatibility, biodegradability, immune evasion properties, and functionality [9,10,11]. EVs, renowned for their nano size, are released from various cancer cells [11,12,13,14]. Paradoxically, these particles can be repurposed for the design of antitumor drug carriers through their extraction and purification [11,13,14]. Given the considerable interest in the development of stimuli-responsive drug carriers, aimed at preventing abnormal drug distribution to normal tissues and enhancing drug accumulation in the target tumor site, stimuli-responsive EVs prepared through a simple encoding method using functional adjuvants may prove suitable for efficient tumor therapy [11,15,16]. These systems are anticipated to exhibit favorable responses to environmental pH stimuli, promptly initiating drug release upon the onset of the desired effect.

**Figure 1:**
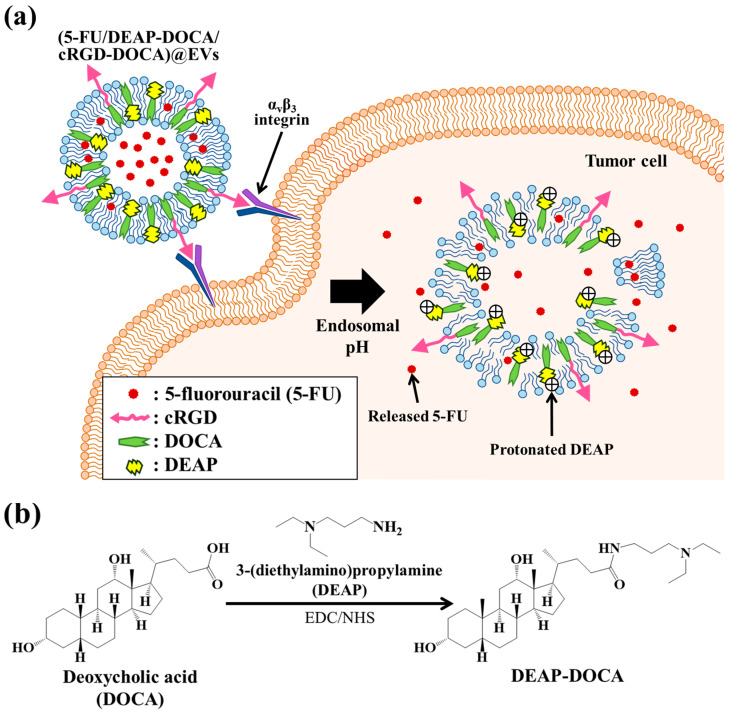

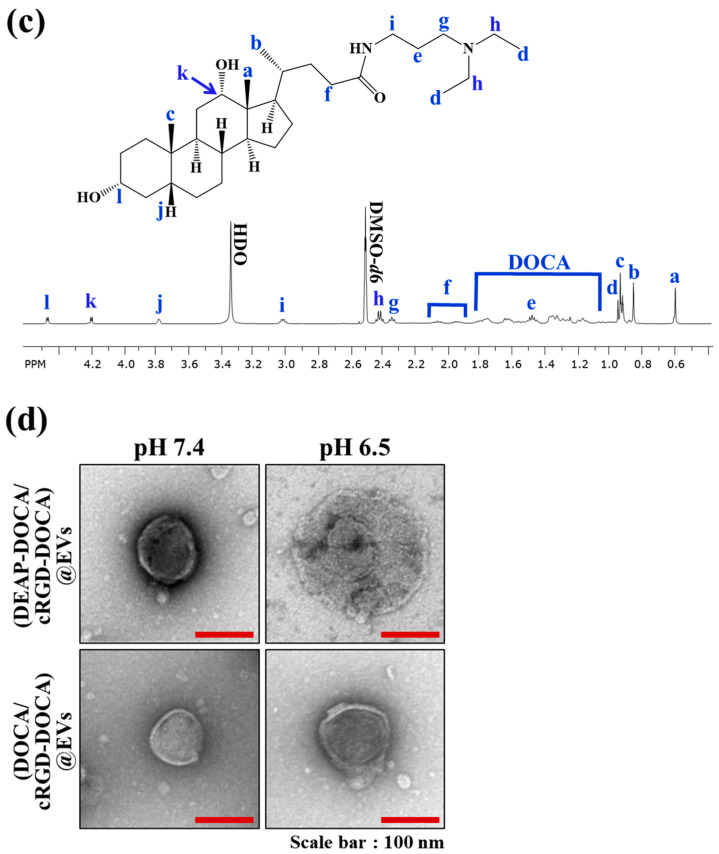
(**a**) Schematic concept presentation of (5-FU/DEAP-DOCA/cRGD-DOCA)@EVs. (**b**) Chemical synthesis scheme of DEAP-DOCA. (**c**) ^1^H-NMR peaks of DEAP-DOCA. (**d**) The enlarged TEM images of (DEAP-DOCA/cRGD-DOCA)@EVs and (DOCA/cRGD-DOCA)@EVs at pH 7.4 and 6.5.

In this study, we engineered pH-responsive antitumor extracellular vesicles (EVs) utilizing a pH-responsive adjuvant, 5-fluorouracil (5-FU, as a model antitumor drug [17,18,19]), and cyclic arginine-glycine-aspartic acid peptide (cRGD [20,21,22,23,24,25], as a tumor-targeting ligand) (Figure 1a). Initially, a pH-responsive adjuvant was synthesized through the chemical coupling of 3-(diethylamino)propylamine (DEAP [26,27,28,29]) to deoxycholic acid (DOCA) (Figure 1b). This pH-responsive adjuvant (DEAP-DOCA) incorporates DEAP (pK_b_ ~ 7.0) [27,28], undergoing protonation/deprotonation based on the pH levels. When DEAP-DOCA is incorporated into the membrane of EVs, it undergoes protonation at an acidic pH, resulting in the destabilization of the vesicle membrane [27,30]. Conversely, at a normal pH of 7.4, it remains non-protonated, contributing to the stability of the vesicle membrane (Figure 1a). Therefore, we anticipate that EVs encoded with 5-FU and DEAP-DOCA will undergo destabilization at an endosomal pH, releasing 5-FU within the tumor cells and enhancing the antitumor efficacy. In addition, cRGD was incorporated into EVs to target α_v_β_3_ integrin receptors on tumor cells [31,32,33]. Based on this hypothesis, we expect that this functional EVs system will demonstrate selectively enhanced the antitumor efficacy at tumor sites.

## 2. Materials and Methods

### 2.1. Materials

Deoxycholic acid (DOCA), 3-(diethylamino)propylamine (DEAP), dimethylsulfoxide (DMSO), *N*-(3-dimethylaminopropyl)-*N*′-ethylcarbodiimide hydrochloride (EDC), *N*-hydroxysuccinimide (NHS), *N*-(2-aminoethyl)maleimide (AEM) trifluoroacetate salt, 5-fluorouracil (5-FU), 4′,6-diamidino-2-phenylindole dihydrochloride (DAPI), and paraformaldehyde were purchased from Sigma-Aldrich (St. Louis, MO, USA). Cyclic arginine-glycine-aspartic acid (cRGD) and TAMRA-labeled cRGD were purchased from Peptron, Inc. (Daejeon, Republic of Korea). Phosphate-buffered saline (PBS), Dulbecco’s modified Eagle’s medium (DMEM), penicillin, streptomycin, fetal bovine serum (FBS), trypsin, and ethylene diamine tetra-acetic acid (EDTA) were purchased from Welgene Inc. (Seoul, Republic of Korea). EV-depleted FBS was purchased from System Biosciences Inc. (Palo Alto, CA, USA). The Micro BCA™ Protein Assay Kit and 1,1′-dioctadecyl-3,3,3′,3′-tetramethylindotricarbocyanine iodide (DiR) were purchased from Thermo Fisher Scientific Inc. (Waltham, MA, USA). The total Bile Acid Assay Kit was purchased from Cell Biolabs, Inc. (San Diego, CA, USA). The Cell Counting Kit-8 (CCK-8) was purchased from Dojindo Molecular Technologies Inc. (Rockville, MD, USA). 1,1′-Dioctadecyl-3,3,3′,3′-tetramethylindodicarbocyanine and 4-chlorobenzenesulfonate salt (DiD) were purchased from Biotium, Inc. (Fremont, CA, USA). Wheat Germ Agglutinin Alexa Fluor^®^ 488 conjugate (WGA-Alexa Fluor^®^ 488) was purchased from Life Technologies (Carlsbad, CA, USA).

### 2.2. DEAP-DOCA and cRGD-DOCA Synthesis

DEAP-DOCA was synthesized by reacting 200 mg of DOCA and 199 mg of DEAP in 16 mL of DMSO containing 293 mg of EDC and 176 mg of NHS at 25 °C for 3 days (Figure 1b). Following the reaction, the solution was added to distilled water to collect the precipitated DEAP-DOCA. The resulting solution was ultracentrifuged at 100,000× *g* for 1 h at 4 °C to remove any unreacted substances. The DEAP-DOCA pellet was suspended in distilled water and ultracentrifuged at 100,000× *g* for 1 h at 4 °C. Subsequently, the supernatant was removed to eliminate any unreacted EDC, NHS, and DEAP. The purified DEAP-DOCA was suspended in distilled water and freeze-dried, yielding DEAP-DOCA. Next, cRGD-DOCA was synthesized using the thiol–maleimide [24,25,34] click reaction (Figure S1). Initially, 50 mg of DOCA was reacted with 324 mg of AEM in 14 mL of DMSO containing 122 mg of EDC and 73 mg of NHS at 25 °C for 3 days. The solution was then added to distilled water to collect the precipitated DOCA-AEM. The resulting solution was ultracentrifuged at 100,000× *g* for 1 h at 4 °C to remove any unreacted substances. The resulting pellet of DOCA-AEM was resuspended in distilled water, ultracentrifuged, and freeze-dried. Subsequently, cRGD-DOCA (1 mg/mL) was synthesized after a chemical reaction between the thiol group of TAMRA-labeled cRGD and the maleimide group of DOCA-AEM at a 1:1 molar ratio in 1 mL of DMSO at 25 °C for 4 h. The unreacted substances were removed through a dialysis process [24].

### 2.3. Isolation of EVs

The mouse macrophage RAW 264.7 cells, obtained from the Korean Cell Line Bank (Seoul, Republic of Korea), were cultured in DMEM supplemented with 1% penicillin–streptomycin and 10% EV-depleted FBS in a 5% CO_2_ atmosphere at 37 °C. During the cell incubation, the supernatant containing the EVs was centrifuged at 2000× *g* for 20 min and then at 100,000× *g* for 30 min to eliminate any dead cells and cell debris [30,35,36,37,38]. Subsequently, the supernatant was ultracentrifuged at 100,000× *g* at 4 °C for 70 min to obtain an EV pellet. The pellet was further purified by washing with PBS and subjected to ultracentrifugation at 100,000× *g* at 4 °C for 70 min. The obtained EVs were quantified using the BCA^TM^ Protein Assay Kit [27,30]. In addition, the MISEV2023 recommendations offer methodologies for the production, isolation, and various characterization aspects related to EVs, which we have partially employed [39].

### 2.4. Preparation of EV Samples

The EVs (500 μg), dispersed in PBS (pH 7.4, 150 mM, 5 mL), were encoded with DOCA derivatives [DEAP-DOCA (250 μg) and/or cRGD-DOCA (50 μg) dissolved in DMSO (100 μL)] and 5-FU [1 mg, dissolved in 1 mL of PBS (pH 7.4, 150 mM)] via sonication using a tip sonicator (vcx-130 with cv-18, Sonics, Newtown, CT, USA) at 30% amplitude, with a cycle of 30 s on and 150 s off, repeated for 6 cycles. Subsequently, the EVs were incubated at 37 °C in a water bath for 1 h, and then some aggregates were removed by filtration using a 0.22 μm filter [30,37]. The resulting solution underwent ultracentrifugation at 100,000× *g* at 4 °C for 70 min to eliminate any free cRGD-DOCA, DEAP-DOCA, and 5-FU. Finally, we prepared (5-FU/DEAP-DOCA/cRGD-DOCA)@EVs, (5-FU/DOCA/cRGD-DOCA)@EVs (without DEAP), (5-FU/DEAP-DOCA)@EVs (without cRGD-DOCA), (5-FU/DOCA)@EVs (without DEAP and cRGD), and (5-FU)@EVs (as a control group). The production yields of the EVs ranged from 50% to 62% by weight.

### 2.5. Characterization of the EV Samples

To assess the encapsulation efficiency of 5-FU within the EVs, the supernatant obtained from ultracentrifugation at 100,000× *g* for 70 min at 4 °C during the EV sample preparation was spectrophotometrically analyzed at 266 nm using a UV-1200 Spectrophotometer (Labentech, Incheon, Republic of Korea) [38,39]. To evaluate the content of TAMRA-labeled cRGD-DOCA in the EVs, the EVs were solubilized in DMSO/PBS (90/10, vol.%) and analyzed using a microplate reader (Bio-Tek, Winooski, VT, USA) at λ_ex_ 557 nm and λ_em_ 583 nm [37,40]. The encoded DEAP-DOCA content was determined by analyzing the DOCA levels in the supernatant during the EVs’ encoding process [41]. In addition, the encoded DEAP-DOCA content was calculated inversely based on the total amount of DOCA in the supernatant using the Total Bile Acid Assay Kit [42]. Here, the loading efficiency (%) of 5-FU, DEAP-DOCA, and cRGD-DOCA in the EVs was calculated as the weight percentage of the encoded substance relative to the initial dosage. The loading content (%) of 5-FU, DEAP-DOCA, and cRGD-DOCA was calculated as the weight percentage of each substance in the EVs [30,37,41].

Next, we examined the morphologies of the EV samples at pH 7.4 (normal pH) and pH 6.5 (endosomal pH) using a transmission electron microscope (TEM, JEOL, Tokyo, Japan) [30,37]. The particle size and zeta potential of the EV samples (50 μg/mL) dispersed in 150 mM PBS (pH 7.4, pH 7.0, and pH 6.5) were determined using a Zetasizer 3000 (Malvern Instruments, Malvern, UK) [30,37]. Additionally, to assess the stability of the EVs, the EV samples (50 μg/mL) were incubated at 37 °C in 150 mM PBS (pH 7.4) for 7 days, and their average particle size was monitored.

### 2.6. In Vitro 5-FU Release Test

The release kinetics of 5-FU from the EV samples were assessed at both pH 7.4 and pH 6.5. Briefly, the EVs (equivalent to 5-FU 100 μg/mL) dispersed in 2 mL of 150 mM PBS (at pH 7.4 and 6.5) were placed inside a dialysis membrane (Spectra/Por^®^ MWCO 50 K). The resulting dialysis membrane bag was sealed and submerged in a fresh 15 mL of 150 mM PBS (at pH 7.4 and 6.5). The 5-FU release experiments were conducted using a mechanical shaker (100 rpm) at 37 °C for 48 h. At various time intervals, samples of PBS (15 mL) were collected from the outer side of the dialysis membrane, and fresh PBS (15 mL) was replenished. The quantity of 5-FU released from the EVs was quantified using a UV-1200 Spectrophotometer (Labentech, Incheon, Republic of Korea) at 266 nm [38,39].

### 2.7. Cell Culture

The murine melanoma B16BL6 cells (integrin α_v_β_3_-positive, passage number: 70) and murine colorectal carcinoma CT-26 cells (integrin α_v_β_3_-negative, passage number: 59), purchased from the Korean Cell Line Bank (Seoul, Republic of Korea), were cultured in DMEM supplemented with 1% penicillin–streptomycin and 10% FBS in an atmosphere of 5% CO_2_ at 37 °C [43]. In addition, we conducted our experiments in a sterilized environment and assessed the possibility of cell line contamination through microscopic examination. 

### 2.8. In Vitro Cytotoxicity Test

The B16BL6 and CT-26 tumor cells were cultured with the EV samples (equivalent to 5-FU 10 μg/mL) or free 5-FU (10 μg/mL) in DMEM at 37 °C for 24 h. The viability of the tumor cells was assessed using the CCK assay. Furthermore, to investigate the toxicity of the drug-free EV samples, the B16BL6 and CT-26 tumor cells were exposed to drug-free EV samples (1 × 10^7^ to 1 × 10^9^ particles/mL) at 37 °C for 24 h. Cell viability was determined using the CCK-8 assay [24,25,30,37].

### 2.9. In Vitro Cellular Uptake Test

To visualize the cellular uptake of each EV sample, the EVs were labeled with DiD dye. Briefly, the EV samples were incubated with DiD dye (1 mM) at 37 °C for 24 h. The solution was then centrifuged at 100,000× *g* for 30 min at 4 °C, and the supernatant was further ultracentrifuged at 100,000× *g* for 70 min at 4 °C. Subsequently, the pellet was suspended in 30 mL of 150 mM PBS (pH 7.4) and ultracentrifuged at 100,000× *g* for 70 min at 4 °C to remove any free DiD dye. Next, the B16BL6 and CT-26 tumor cells were incubated with each EV sample (equivalent to DiD 5 μg/mL) at 37 °C for 4 h. Subsequently, the cells were washed three times with PBS (pH 7.4, 150 mM). Additionally, for the visualization of the cell nuclei and cell membranes, the B16BL6 and CT-26 tumor cells were stained using DAPI and WGA-Alexa Fluor^®^488, followed by fixation with 3.7% formaldehyde solution. The fixed cells were analyzed using a confocal laser scanning microscope (Carl Zeiss, LSM710, Oberkochen, Germany) [24,25,30,37].

### 2.10. Animal Care

The in vivo experiments were conducted using 6- to 8-week-old female BALB/c mice (CAnN.Cg-Foxn1 nu/CrlOri) weighing approximately 20 g, purchased from Orient Bio Inc. (Seoul, Republic of Korea). The mice were housed in a controlled environment and all the procedures were performed in accordance with the guidelines of an approved protocol (code: CUK-IACUC-2023-015) from the Institutional Animal Care and Use Committee (IACUC) of the Catholic University of Korea (Republic of Korea) [27].

### 2.11. In Vivo Tumor Inhibition Test

To assess the tumor inhibitory efficacy of the EV samples in vivo, two tumor allograft models were established. The B16BL6 and CT-26 tumor cells (1×10^7^ cells/mL) were subcutaneously implanted into the left thigh and right thigh of BALB/c mice, respectively. When the transplanted tumors reached approximately 100 mm^3^ in volume, the EV samples (equivalent to 5-FU 20 mg/kg), free 5-FU (20 mg/kg), and saline (control) were intravenously administered via the tail vein. The tumor volumes were monitored for 7 days, and the tumor volume was calculated using the following formula: tumor volume = length × (width)^2^/2. The relative change in the tumor volume (V_t_/V_0_, where V_t_ is the tumor volume at a specific time and V_0_ is the initial tumor volume) was plotted [30,37].

### 2.12. In Vivo Biodistribution

To track the biodistribution of the EV samples, we labeled the EVs with in vivo fluorescence DiR dye [37]. Briefly, the EV samples were incubated with DiR dye (1 mM) and cultured at 37 °C for 24 h. The solution was then centrifuged at 100,000× *g* for 30 min at 4 °C, and the supernatant was further ultracentrifuged at 100,000× *g* for 70 min at 4 °C. Subsequently, the pellet was suspended in 30 mL of 150 mM PBS (pH 7.4) and ultracentrifuged at 100,000× *g* for 70 min at 4 °C to remove any free DiR dye. The DiR dye-labeled EV samples (equivalent to DiR 2.0 mg/kg) or free DiR (2.0 mg/kg) were intravenously injected via the tail vein. The mice were analyzed using the Fluorescence-labeled Organism Bioimaging Instrument (FOBI, NeoScience, Seoul, Republic of Korea) for 24 h. At 24 h post-injection, the mice were euthanized using CO_2_ gas. Subsequently, the major organs (liver, heart, lungs, spleen, and kidneys) and tumors were harvested and analyzed using the FOBI. The quantification of the integrated fluorescence intensity was performed using NEOimage instrument (NeoScience, Seoul, Republic of Korea) [25,37].

### 2.13. Statistics

Statistical analysis of all the data was conducted using Student’s *t*-test (nonparametric test) or an analysis of variance (ANOVA) at a significance level of *p* < 0.01 (**) [25,27].

## 3. Results and Discussion

### 3.1. Fabrication of the EV Samples

To synthesize pH-responsive EVs targeting α_v_β_3_ integrin receptors on tumor cells (Figure 1a), we isolated EVs from RAW264.7 cells and integrated DOCA derivatives (DEAP-DOCA and cRGD-DOCA), along with 5-FU (a model drug for antitumor activity) [17,18,19,39], into the EVs, resulting in the fabrication of (5-FU/DEAP-DOCA/cRGD-DOCA)@EVs. DEAP-DOCA was synthesized by chemically conjugating DEAP to DOCA in DMSO containing EDC and NHS (Figure 1b). The resulting DEAP-DOCA was analyzed using ^1^H NMR, where we characterized the integration ratio of the peaks at δ 2.42 ppm (-CH_2_- from DEAP) and δ 4.20 ppm (-CH from DOCA) (Figure 1c). Consequently, the molar conjugation ratio of DEAP to DOCA was determined to be 1:1. Furthermore, cRGD-DOCA was prepared by chemically coupling DOCA (preactivated using AEM in DMSO containing EDC and NHS) with the cRGD peptide through the thiol–maleimide click reaction (Figure S1a). Through characterization of the integration ratio of the ^1^H NMR peaks at δ 6.51 ppm (-CH- from cRGD) and δ 4.20 ppm (-CH from DOCA), the molar conjugation ratio of cRGD to DOCA was assumed to be 1:1 (Figure S1b). Subsequently, 5-FU, DEAP-DOCA, and cRGD-DOCA were incorporated into the EVs using the sonication method. We prepared various formulations, including (5-FU/DEAP-DOCA/cRGD-DOCA)@EVs, (5-FU/DOCA/cRGD-DOCA)@EVs (without DEAP), (5-FU/DEAP-DOCA)@EVs (without cRGD-DOCA), (5-FU/DOCA)@EVs (without DEAP and cRGD), and (5-FU)@EVs (as a control group). The loading efficiency of the 5-FU, DEAP-DOCA (or DOCA), and cRGD-DOCA in the EV samples ranged from approximately 8.4% to 9.0% by weight, 42.4% to 43.0% by weight, and 28.0% to 34.0% by weight, respectively. The loading content of the 5-FU, DEAP-DOCA (or DOCA), and cRGD-DOCA in the EV samples ranged from approximately 16.5% to 18.4% by weight, 21.1% to 21.8% by weight, and 2.9% to 3.3% by weight, respectively.

### 3.2. Characterization of the EV Samples

Figure 1d depicts TEM images of the EV samples at pH 7.4 (normal pH) and pH 6.5 (endosomal pH). At pH 7.4, both the (DEAP-DOCA/cRGD-DOCA)@EVs and (DOCA/cRGD-DOCA)@EVs displayed similar vesicle morphologies and maintained stable vesicle membrane structures. However, at pH 6.5, the (DEAP-DOCA/cRGD-DOCA)@EVs became destabilized, exhibiting an unstable membrane structure. These findings indicate that the DEAP component (with a pK_b_ ~ 7.0) of DEAP-DOCA influences the stability of the vesicle membrane.

**Figure 2:**
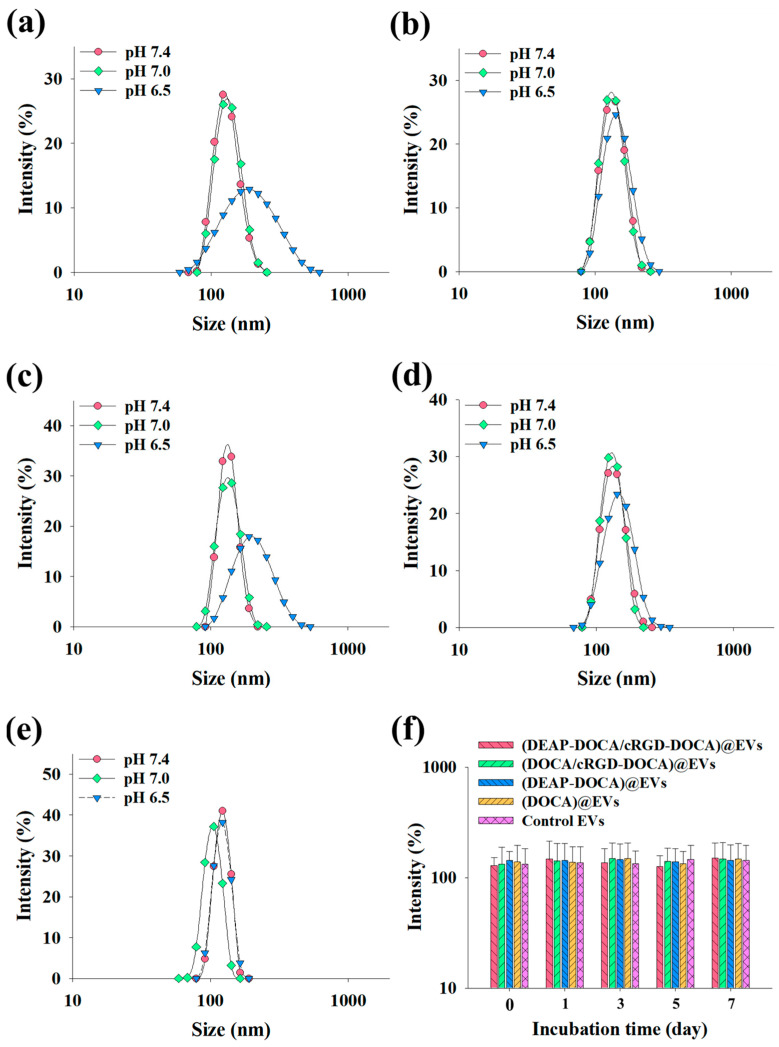
Particle size distribution of (**a**) (DEAP-DOCA/cRGD-DOCA)@EVs, (**b**) (DOCA/cRGD-DOCA)@EVs, (**c**) (DEAP-DOCA)@EVs, (**d**) (DOCA)@EVs, (**e**) control EVs at pH 7.4, 7.0 and 6.5 (n = 3, as multiple experiments). (**f**) Average particle size of each EV sample incubated in PBS (150 mM, pH 7.4) for 7 days at 37 °C (n = 3, as multiple experiments).

**Figure 3:**
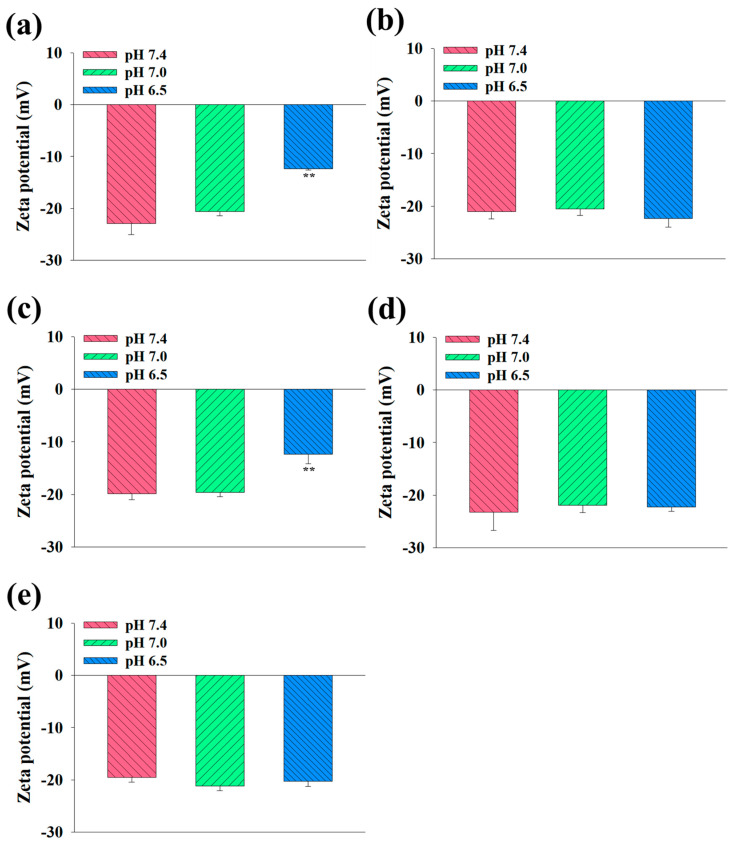
Zeta potential values of (**a**) (DEAP-DOCA/cRGD-DOCA)@EVs, (**b**) (DOCA/cRGD-DOCA)@EVs, (**c**) (DEAP-DOCA)@EVs, (**d**) (DOCA)@EVs, and (**e**) control EVs at pH 7.4, 7.0 and 6.5 (n = 3, as multiple experiments, ** *p* < 0.01 compared to pH 7.4).

Next, we assessed the average particle size of the EV samples at pH 7.4, 7.0, and 6.5 utilizing a Zetasizer 3000. As depicted in Figure 2a–e, the average particle size of the (DEAP-DOCA/cRGD-DOCA)@EVs and (DEAP-DOCA)@EVs was measured as 132 nm (polydispersity index: 0.39 ± 0.09) and 136 nm (polydispersity index: 0.54 ± 0.12) at pH 7.4, respectively, and 131 nm (polydispersity index: 0.28 ± 0.01) and 136 nm (polydispersity index: 0.30 ± 0.04) at pH 7.0. Notably, these sizes escalated to 206 nm (polydispersity index: 0.21 ± 0.01) and 212 nm (polydispersity index: 0.29 ± 0.07) at pH 6.5, attributed to the protonation of DEAP, consequently enlarging the size of the EVs. Conversely, the (DOCA/cRGD-DOCA)@EVs, (DOCA)@EVs, and control EVs exhibited negligible size alterations across the pH levels (pH 7.4, 7.0, and 6.5). Furthermore, the stability of the EV samples was verified, as their sizes in PBS (pH 7.4, 150mM) ranged from 128 (polydispersity index: 0.30 ± 0.03) to 142 nm (polydispersity index: 0.27 ± 0.03) on day 0, akin to 142 (polydispersity index: 0.14 ± 0.01) to 150 nm (polydispersity index: 0.17 ± 0.09) on day 7 of the incubation (Figure 2f). Figure 3 shows the zeta potential variations of the EV samples across the different pH levels. The zeta potentials of the (DEAP-DOCA/cRGD-DOCA)@EVs and (DEAP-DOCA)@EVs surged from −22.9 and −19.8 mV to −12.4 and −12.3 mV, respectively, with a decreasing pH from 7.4 to 6.5. Nevertheless, there existed no significant disparity in the zeta potential of the (DOCA/cRGD-DOCA)@EVs, (DOCA)@EVs, and control EVs at pH 7.4, 7.0, and 6.5. These findings demonstrate that at pH 6.5, the protonated DEAP within DEAP-DOCA instigated the destabilization of the vesicle membrane structures, thereby inducing alterations in their particle size and zeta potential.

### 3.3. In Vitro 5-FU Release of the EV Samples

**Figure 4:**
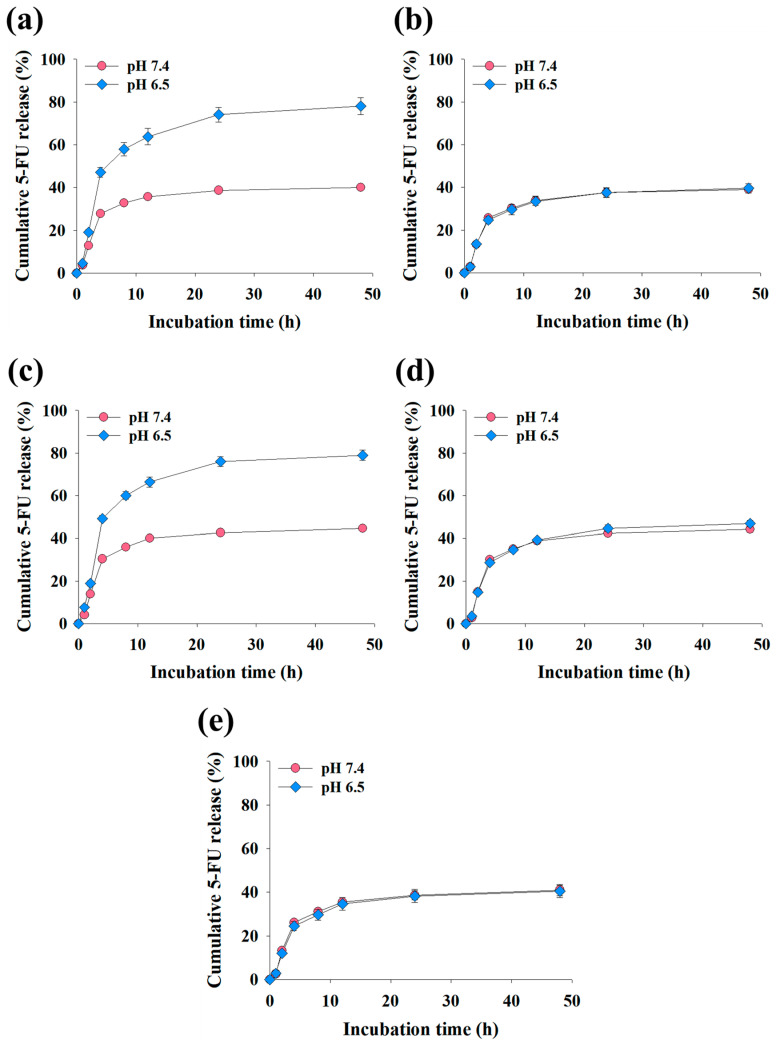
Cumulative 5-FU release profile of (**a**) (5-FU/DEAP-DOCA/cRGD-DOCA)@EVs, (**b**) (5-FU/DOCA/cRGD-DOCA)@EVs, (**c**) (5-FU/DEAP-DOCA)@EVs, (**d**) (5-FU/DOCA)@EVs, and (**e**) (5-FU)@EVs for 48 h (n = 3, as multiple experiments).

The cumulative 5-FU release profiles of the EV samples under various pH conditions are illustrated in Figure 4. Notably, the (5-FU/DEAP-DOCA/cRGD-DOCA)@EVs and (5-FU/DEAP-DOCA)@EVs exhibited a release of 40–45 wt % of 5-FU at pH 7.4, whereas at pH 6.5 [30,41], they displayed an almost twofold increase, releasing 78–79 wt % of 5-FU after 48 h of incubation. Furthermore, their 5-FU release rate was significantly augmented at pH 6.5 in comparison to the (5-FU/DOCA/cRGD-DOCA)@EVs (Figure 4a–c). However, the EV samples lacking DEAP-DOCA showed a similar release trend at both pH 7.4 and pH 6.5, releasing 39–44 wt % of 5-FU and 40–47 wt % after 48 h of incubation (Figure 4b,d,e). These findings indicate that at pH 6.5 (endosomal pH) [30,41], the protonation of DEAP in DEAP-DOCA expedites the release of 5-FU from (5-FU/DEAP-DOCA/cRGD-DOCA)@EVs and (5-FU/DEAP-DOCA)@EVs. 

### 3.4. In Vitro Cell Cytotoxicity of the EV Samples

**Figure 5:**
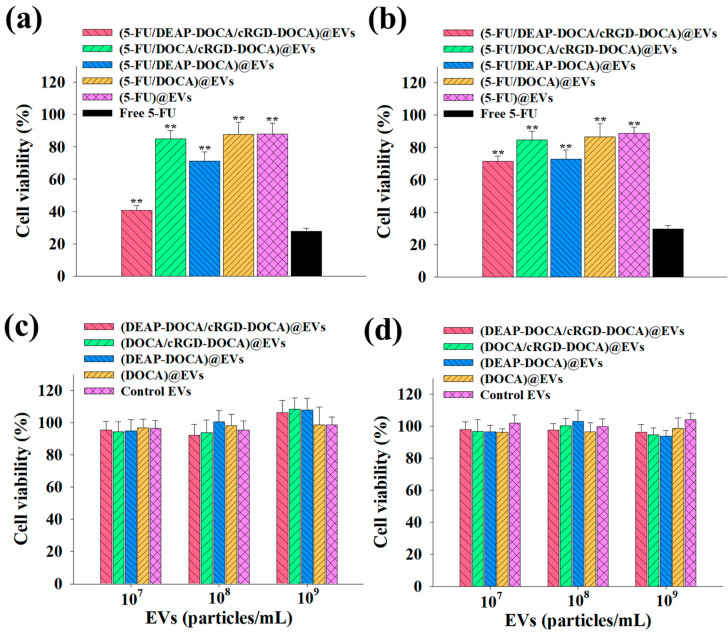

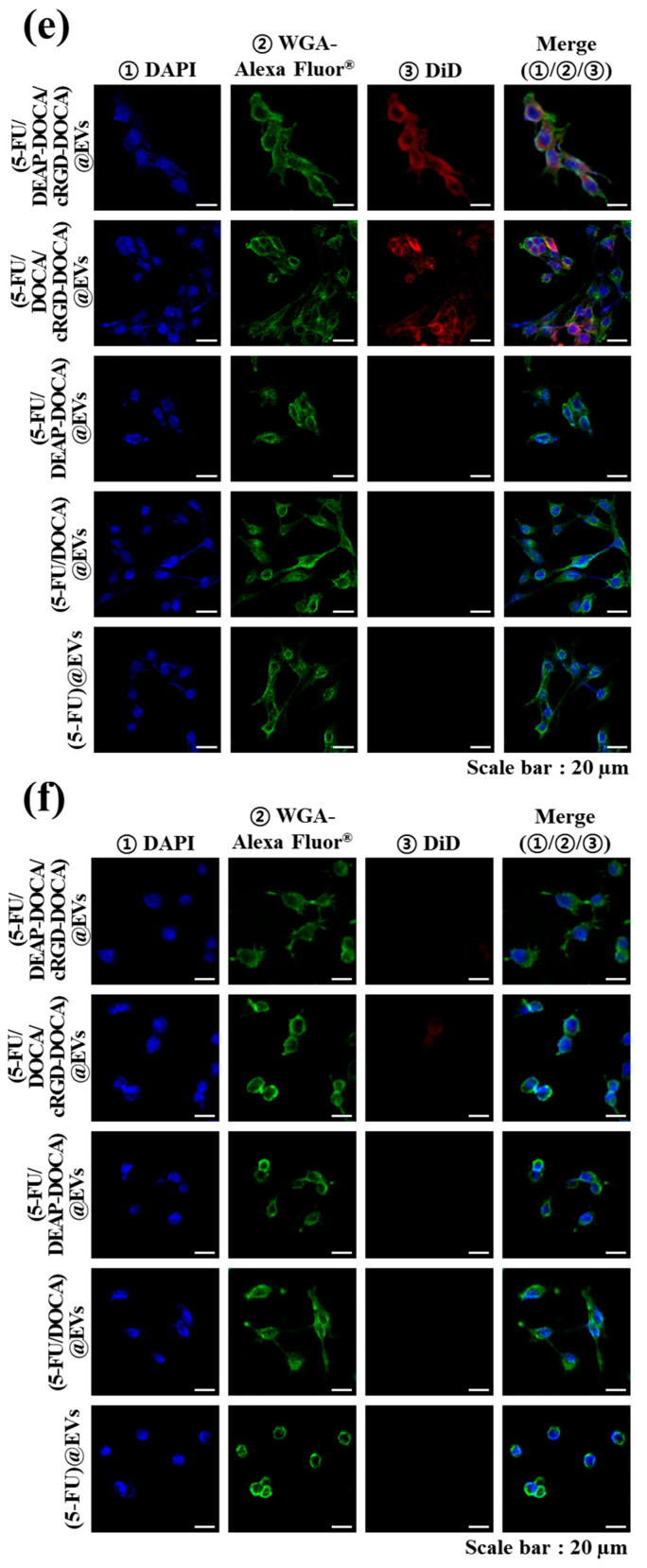
In vitro cell viabilities determined by the Cell Counting Kit-8 (CCK-8) assay of (**a**) B16BL6 (integrin α_v_β_3_-positive) and (**b**) CT-26 cells (integrin α_v_β_3_-negative) treated with each sample (equivalent to 5-FU 10 μg/mL) or free 5-FU (10 μg/mL) for 24 h at 37 °C (n = 7, as multiple experiments, ** *p* < 0.01 compared to free 5-FU). Cell viability of (**c**) B16BL6 and (**d**) CT-26 cells treated with each EV sample (without 5-FU) for 24 h at 37 °C (n = 7, as multiple experiments, ** *p* < 0.01 compared to the control EVs). Confocal microscope images of (**e**) B16BL6 and (**f**) CT-26 cells treated with each EV sample (equivalent to DiD 5 μg/mL) for 4 h at 37 °C.

To assess the cell cytotoxicity and cellular uptake behaviors of the EVs, we employed cells with varying origins and α_v_β_3_ integrin expressions; specifically, cells harboring α_v_β_3_ integrin (B16BL6 cells) [44] and cells devoid of it (CT26 cells) [45]. The tumor cells were exposed to the EV samples (equivalent to 5-FU 10 μg/mL) or free 5-FU (10 μg/mL). The results showed that the cell viabilities of the B16BL6 cells treated with the EV samples lacking DEAP-DOCA or cRGD-DOCA were above 70%. However, the (5-FU/DEAP-DOCA/cRGD-DOCA)@EVs reduced the cell viability of the B16BL6 tumor cells to 40.6%, likely due to both the cRGD/α_v_β_3_ integrin receptor-mediated endocytosis [20,24,25] and the endosomal pH-responsive [25,27,30,41] 5-FU release (Figure 5a). However, these EV samples exhibited lower cytotoxicity toward the CT26 (integrin α_v_β_3_-negative) tumor cells (Figure 5b). Furthermore, by incubating the B16BL6 and CT-26 cells with the EV samples lacking 5-FU, we evaluated the intrinsic toxicity, confirming that the cytotoxicities of the EV samples without 5-FU were not significant (Figure 5c,d). In addition, free 5-FU does not demonstrate tumor cell-specific toxicity for each individual tumor cell, as it shows aggressiveness toward both types of cells. It is known that free 5-FU is toxic to even normal cells. Our designed EVs, targeting α_v_β_3_ integrin, may mitigate the side effects by specifically targeting tumor cells expressing α_v_β_3_ integrin. Naturally, further investigation into more specific aspects is warranted in the future. We also noted that the EV samples with cRGD-DOCA showed relatively high cellular uptake by the B16BL6 (integrin α_v_β_3_-positive) tumor cells (Figure 5e), which is comparable to the low cellular uptake of the EV samples with cRGD-DOCA in the CT26 (integrin α_v_β_3_-negative) tumor cells (Figure 5f). Here, all the EV samples were labeled with a fluorescent DiD dye for visualization [46]. Moreover, the (5-FU/DEAP-DOCA/cRGD-DOCA)@EVs (without a fluorescent DiD dye) exhibited no fluorescent intensity in the B16BL6 tumor cells (Figure S2). The percentage of DiD dye labeled per 1 mg of each EV sample was approximately 0.02 mg. To quantify the DiD dye labeled on the EVs, the EVs were solubilized in DMSO/PBS (90/10, vol.%) and analyzed using a microplate reader (Bio-Tek, Winooski, VT, USA) at λ_ex_ 644 nm and λ_em_ 663 nm [37,47].

### 3.5. In Vivo Tumor Inhibition of the EV Samples

**Figure 6:**
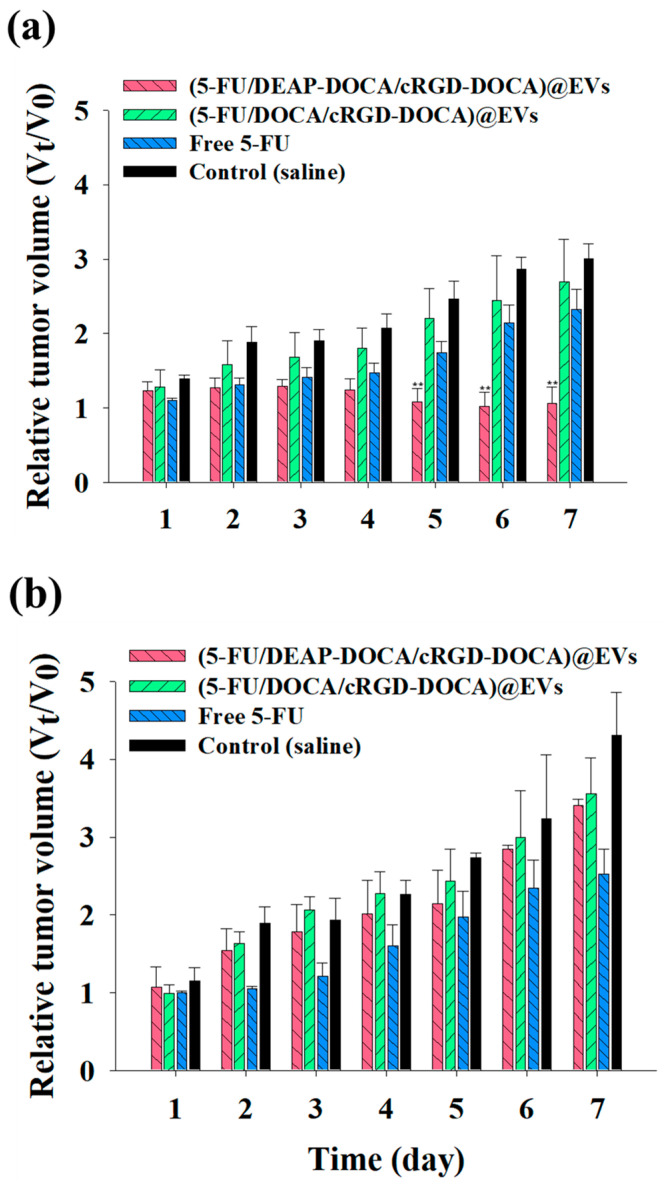
Relative tumor volume change (V_t_/V_0_) of (**a**) B16BL6 and (**b**) CT-26 tumor-bearing BALB/c mice intravenously injected with each EV sample (equivalent to 5-FU 20 mg/kg), free 5-FU (20 mg/kg), or saline (control) (n = 3, as multiple experiments, ** *p* < 0.01 compared to free 5-FU).

The therapeutic efficacy of the EV samples was investigated in B16BL6 (at the left thigh)/CT-26 (at the right thigh) tumor-bearing mice (Figure 6). Here, the (5-FU/DEAP-DOCA/cRGD-DOCA)@EVs (equivalent to 5-FU 20 mg/kg), (5-FU/DOCA/cRGD-DOCA)@EVs (equivalent to 5-FU 20 mg/kg), and free 5-FU (20 mg/kg) were intravenously administered, and the tumor volumes were monitored for 7 days. At 7 days post-injection, the relative B16BL6 tumor volume in the mice treated with (5-FU/DEAP-DOCA/cRGD-DOCA)@EVs was approximately 2.5, 2.2, and 2.8 times smaller than those in the mice treated with (5-FU/DOCA/cRGD-DOCA)@EVs, free 5-FU, and control (saline), respectively (Figure 6a). However, the relative CT-26 tumor volume in the mice treated with (5-FU/DEAP-DOCA/cRGD-DOCA)@EVs was almost 1.3 times larger than that in the mice treated with free 5-FU. There was no significant difference in the CT-26 tumor volume between the mice treated with (5-FU/DEAP-DOCA/cRGD-DOCA)@EVs, (5-FU/DOCA/cRGD-DOCA)@EVs, and control (saline) (Figure 6b). These results suggest that (5-FU/DEAP-DOCA/cRGD-DOCA)@EVs can effectively inhibit B16BL6 tumor growth in vivo, as demonstrated in the in vitro cytotoxicity test (Figure 5a).

### 3.6. In Vivo Biodistribution of the EV Samples

**Figure 7:**
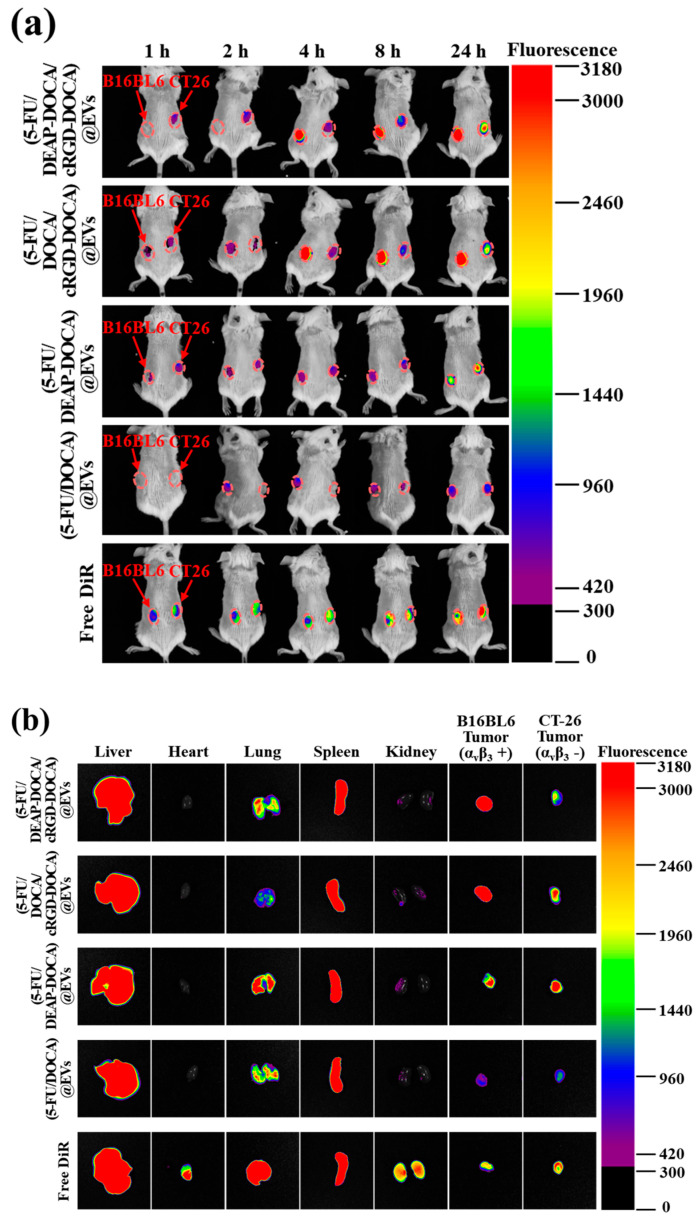

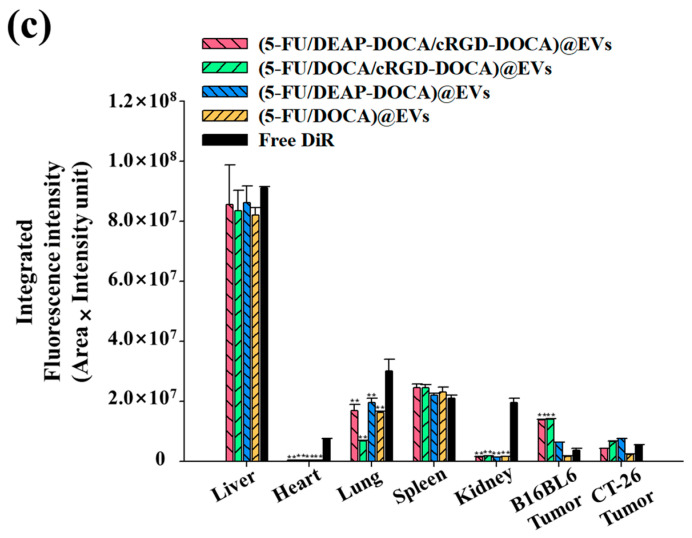
(**a**) In vivo fluorescence images of DiR dye-labeled EV samples intravenously injected into B16BL6/CT-26 tumor-bearing BALB/c mice. Fluorescence images were obtained for 24 h post-injection. (**b**) The fluorescence images of major organs and tumors harvested from B16BL6/CT-26 tumor-bearing BALB/c mice at 24 h post-injection of each EV sample. (**c**) Integrated fluorescence intensity of major organs and tumors (n = 3, ** *p* < 0.01 compared to free DiR).

To evaluate the in vivo tumor-targeting efficacy of the EV samples, we labeled them with a fluorescent DiR dye. The amount of DiR dye labeled per 1 mg of each EV sample was approximately 0.02 mg. The DiR dye labeled on EVs was assessed after solubilizing the EVs in DMSO/PBS (90/10, vol.%) and by analyzing them using a microplate reader (Bio-Tek, Winooski, VT, USA) at λ_ex_ 748 nm and λ_em_ 780 nm [37,48]. Each EV sample (equivalent to DiR 2.0 mg/kg) and free DiR (2.0 mg/kg) were intravenously administered to B16BL6/CT-26 tumor-bearing mice, and the fluorescence was monitored for 24 h using the FOBI (Figure 7). At 4 h post-injection, strong fluorescence signals were observed at the B16BL6 tumor sites for the (5-FU/DEAP-DOCA/cRGD-DOCA)@EVs and (5-FU/DOCA/cRGD-DOCA)@EVs. However, the fluorescence signals from the other samples were relatively weak, possibly due to the lower accumulation of EV samples without cRGD-DOCA. At 24 h post-injection, to further confirm the biodistribution of the EV samples, we acquired fluorescence images of the major organs (liver, heart, lungs, spleen, and kidneys) and tumors. Free DiR was evenly distributed in all the major organs and tumors, while the (5-FU/DEAP-DOCA/cRGD-DOCA)@EVs and (5-FU/DOCA/cRGD-DOCA)@EVs were primarily concentrated in the organs (liver and spleen) associated with the reticuloendothelial system [49] and tumors (Figure 7b). As depicted in Figure 7c, the (5-FU/DEAP-DOCA/cRGD-DOCA)@EVs and (5-FU/DOCA/cRGD-DOCA)@EVs exhibited 3.3-fold and 2.1-fold higher integrated fluorescence intensity in the B16BL6 tumors compared to the CT-26 tumors, respectively. Moreover, in the B16BL6 tumors, they demonstrated 2.2-fold, 8.7-fold, and 3.8-fold higher intensity than the (5-FU/DEAP-DOCA)@EVs, (5-FU/DOCA)@EVs, and free DiR, respectively, indicating that cRGD-DOCA enhances the targeting efficiency of EVs for B16BL6 tumors expressing α_v_β_3_ integrin receptors. Collectively, the (5-FU/DEAP-DOCA/cRGD-DOCA)@EVs incorporating DEAP-DOCA and cRGD-DOCA exhibited significant antitumor efficacy (Figure 6a). Overall, this EV system, derived from cells, is expected to exhibit excellent biocompatibility and biofunctionality compared to conventional liposome-based systems [32,33]. Specifically, in this study, functionalization of the EV system was accomplished using pH-responsive adjuvants, indicating that such systems may provide diverse therapeutic modalities for future tumor treatments. Moreover, the methods utilized here to target α_v_β_3_ integrin show promise for pertinent tumor therapies [36,37,38].

## 4. Conclusions

In this study, we fabricated (5-FU/DEAP-DOCA/cRGD-DOCA)@EVs by integrating EVs derived from RAW264.7 with 5-FU, DEAP-DOCA, and cRGD-DOCA. Both the in vitro and in vivo experimental results demonstrated the enhanced 5-FU release ability of the (5-FU/DEAP-DOCA/cRGD-DOCA)@EVs at an endosomal pH, facilitated by DEAP-DOCA (a pH-responsive adjuvant), along with the selective targeting ability toward α_v_β_3_ integrin receptors via cRGD-DOCA, culminating in outstanding in vivo tumor suppression. Based on these results, these functional EVs hold promise as a compelling approach for tumor treatment, offering selective targeting of specific tumors and prompt drug release. Nonetheless, further validation through experiments involving human tumor cells and xenograft animal models is imperative.

## Data Availability

Data are contained within the article and Supplementary Materials.

## Supplementary Materials

The following supporting information can be downloaded at: https://www.mdpi.com/article/10.3390/pharmaceutics16050599/s1, Figure S1: (a) Chemical synthesis scheme of cRGD-DOCA. (b) ^1^H-NMR peaks of cRGD-DOCA; Figure S2: Confocal microscope images of B16BL6 cells treated with (5-FU/DEAP-DOCA/cRGD-DOCA)@EVs (without DiD) for 4 h at 37 °C.

## References

[1] Pérez-Herrero E., Fernández-Medarde A. (2015). Advanced targeted therapies in cancer: Drug nanocarriers, the future of chemotherapy. Eur. J. Pharm. Biopharm..

[2] Debele T.A., Peng S., Tsai H.C. (2015). Drug carrier for photodynamic cancer therapy. Int. J. Mol. Sci..

[3] Zhang J., Lan C.Q., Post M., Simard B., Deslandes Y., Hsieh T.H. (2006). Design of nanoparticles as drug carriers for cancer therapy. Cancer Genom. Proteom..

[4] Jin K.T., Lu Z.B., Chen J.Y., Liu Y.Y., Lan H.R., Dong H.Y., Yang F., Zhao Y.Y., Chen X.Y. (2020). Recent trends in nanocarrier-based targeted chemotherapy: Selective delivery of anticancer drugs for effective lung, colon, cervical, and breast cancer treatment. J. Nanomater..

[5] Salatin S., Maleki Dizaj S., Yari Khosroushahi A. (2015). Effect of the surface modification, size, and shape on cellular uptake of nanoparticles. Cell Biol. Int..

[6] Hui Y., Yi X., Hou F., Wibowo D., Zhang F., Zhao D., Gao H., Zhao C.X. (2019). Role of nanoparticle mechanical properties in cancer drug delivery. ACS Nano.

[7] Khezri K., Saeedi M., Dizaj S.M. (2018). Application of nanoparticles in percutaneous delivery of active ingredients in cosmetic preparations. Biomed. Pharmacother..

[8] Tsou Y.H., Wang B., Ho W., Hu B., Tang P., Sweet S., Zhang X.Q., Xu X. (2019). Nanotechnology-mediated drug delivery for the treatment of obesity and its related comorbidities. Adv. Healthc. Mater..

[9] Herrmann I.K., Wood M.J.A., Fuhrmann G. (2021). Extracellular vesicles as a next-generation drug delivery platform. Nat. Nanotechnol..

[10] Walker S., Busatto S., Pham A., Tian M., Suh A., Carson K., Quintero A., Lafrence M., Malik H., Santana M.X. (2019). Extracellular vesicle-based drug delivery systems for cancer treatment. Theranostics.

[11] Zhang X., Zhang H., Gu J., Zhang J., Shi H., Qian H., Wang D., Xu W., Pan J., Santos H.A. (2021). Engineered extracellular vesicles for cancer therapy. Adv. Mater..

[12] Becker A., Thakur B.K., Weiss J.M., Kim H.S., Peinado H., Lyden D. (2016). Extracellular vesicles in cancer: Cell-to-cell mediators of metastasis. Cancer Cell.

[13] Liguori G.L., Kralj-Iglič V. (2023). Pathological and therapeutic significance of tumor-derived extracellular vesicles in cancer cell migration and metastasis. Cancers.

[14] Chen J., Fei X., Wang J., Cai Z. (2020). Tumor-derived extracellular vesicles: Regulators of tumor microenvironment and the enlightenment in tumor therapy. Pharmacol. Res..

[15] Tominaga N., Yoshioka Y., Ochiya T. (2015). A novel platform for cancer therapy using extracellular vesicles. Adv. Drug Deliv. Rev..

[16] Bie N., Yong T., Wei Z., Gan L., Yang X. (2022). Extracellular vesicles for improved tumor accumulation and penetration. Adv. Drug Deliv. Rev..

[17] Ghoshal K., Jacob S.T. (1997). An alternative molecular mechanism of action of 5-fluorouracil, a potent anticancer drug. Biochem. Pharmacol..

[18] Longley D.B., Harkin D.P., Johnston P.G. (2003). 5-fluorouracil: Mechanisms of action and clinical strategies. Nat. Rev. Cancer.

[19] Miura K., Kinouchi M., Ishida K., Fujibuchi W., Naitoh T., Ogawa H., Ando T., Yazaki N., Watanabe K., Haneda S. (2010). 5-fu metabolism in cancer and orally-administrable 5-fu drugs. Cancers.

[20] Liu S. (2006). Radiolabeled multimeric cyclic RGD peptides as integrin α_v_β_3_ targeted radiotracers for tumor imaging. Mol. Pharm..

[21] Liolios C., Sachpekidis C., Kolocouris A., Dimitrakopoulou-Strauss A., Bouziotis P. (2021). PET diagnostic molecules utilizing multimeric cyclic RGD peptide analogs for imaging integrin α_v_β_3_ receptors. Molecules.

[22] Danhier F., Le Breton A., Préat V. (2012). RGD-based strategies to target alpha (v) beta (3) integrin in cancer therapy and diagnosis. Mol. Pharm..

[23] Asati S., Pandey V., Soni V. (2019). RGD peptide as a targeting moiety for theranostic purpose: An update study. Int. J. Pept. Res. Ther..

[24] Kim S.K., Lee J.M., Oh K.T., Lee E.S. (2017). Extremely small-sized globular poly (ethylene glycol)-cyclic RGD conjugates targeting integrin α_v_β_3_ in tumor cells. Int. J. Pharm..

[25] Noh G.J., Oh K.T., Youn Y.S., Lee E.S. (2020). Cyclic RGD-conjugated hyaluronate dot bearing cleavable doxorubicin for multivalent tumor targeting. Biomacromolecules.

[26] Duan Z., Gao Y.J., Qiao Z.Y., Fan G., Liu Y., Zhang D., Wang H. (2014). A photoacoustic approach for monitoring the drug release of pH-sensitive poly(β-amino ester)s. J. Mater. Chem. B.

[27] Kang J., Lee E., Lee E.S. (2023). Macrophage membrane-derived pH-responsive nanovesicles to target tumor cells with integrin α4β1 receptor. Macromol. Res..

[28] Kim S.K., Park H., Lee J.M., Na K., Lee E.S. (2018). pH-responsive starch microparticles for a tumor-targeting implant. Polym. Adv. Technol..

[29] Qiao Z.Y., Qiao S.L., Fan G., Fan Y.S., Chen Y., Wang H. (2014). One-pot synthesis of pH-sensitive poly (RGD-co-β-amino ester)s for targeted intracellular drug delivery. Polym. Chem..

[30] Lee H., Park H., Noh G.J., Lee E.S. (2018). pH-responsive hyaluronate-anchored extracellular vesicles to promote tumor-targeted drug delivery. Carbohydr. Polym..

[31] Geng T., Leung E., Chamley L.W., Wu Z. (2023). Functionalisation of extracellular vesicles with cyclic-RGDyC potentially for glioblastoma targeted intracellular drug delivery. Biomater. Adv..

[32] Liu Y., Xia P., Yan F., Yuan M., Yuan H., Du Y., Yan J., Song Q., Zhang T., Hu D. (2023). Engineered Extracellular Vesicles for Delivery of an IL-1 Receptor Antagonist Promote Targeted Repair of Retinal Degeneration. Small.

[33] Wu H., Xing H., Wu M.C., Shen F., Chen Y., Yang T. (2021). Extracellular-vesicles delivered tumor-specific sequential nanocatalysts can be used for MRI-informed nanocatalytic Therapy of hepatocellular carcinoma. Theranostics.

[34] Chen Z., Deng J., Zhao Y., Tao T. (2012). Cyclic RGD peptide-modified liposomal drug delivery system: Enhanced cellular uptake in vitro and improved pharmacokinetics in rats. Int. J. Nanomed..

[35] Chen D., Jiang X. (2022). Exosomes-derived miR-125-5p from cartilage endplate stem cells regulates autophagy and ECM metabolism in nucleus pulposus by targeting SUV38H1. Exp. Cell Res..

[36] Willis M.L., Mahung C., Wallet S.M., Barnett A., Cairns B.A., Coleman Jr L.G., Maile R. (2022). Plasma extracellular vesicles released after severe burn injury modulate macrophage phenotype and function. J. Leukoc. Biol..

[37] Lee E., Lee E.S. (2023). Tumor extracellular vesicles carrying antitumor (KLAKLAK)2 peptide and tumor-specific antigens for improved tumor therapy. J. Pharm. Investig..

[38] Arıca B., Çalış S., Kaş H.S., Sargon M.F., Hıncal A.A. (2002). 5-Fluorouracil encapsulated alginate beads for the treatment of breast cancer. Int. J. Pharm..

[39] Welsh J.A., Goberdhan D.C.I., O’Driscoll L., Buzas E.I., Blenkiron C., Bussolati B., Cai H., Di Vizio D., Driedonks T.A.P., Erdbrügger U. (2024). Minimal information for studies of extracellular vesicles (MISEV2023): From basic to advanced approaches. J. Extracell. Vesicles.

[40] Tolentino M.Q., Hartmann A.K., Loe D.T., Rouge J.L. (2020). Controlled release of small molecules and proteins from DNA-surfactant stabilized metal organic frameworks. J. Mater. Chem. B.

[41] Lee H., Park H., Yu H.S., Na K., Oh K.T., Lee E.S. (2019). Dendritic cell-targeted pH-responsive extracellular vesicles for anticancer vaccination. Pharmaceutics.

[42] Ngoh Y.Y., Choi S.B., Gan C.Y. (2017). The potential roles of Pinto bean (Phaseolus vulgaris cv. Pinto) bioactive peptides in regulating physiological functions: Protease activating, lipase inhibiting and bile acid binding activities. J. Funct. Food.

[43] Choi M.A., Kim S.H., Chung W.Y., Hwang J.K., Park K.K. (2004). Xanthorrhizol, a natural sesquiterpenoid from Curcuma xanthorrhiza, has an anti-metastatic potential in experimental mouse lung metastasis model. Biochem. Biophys. Res. Commun..

[44] Fukuda K., Sugihara E., Ohta S., Izuhara K., Funakoshi T., Amagai M., Saya H. (2015). Periostin is a key niche component for wound metastasis of melanoma. PLoS ONE.

[45] Mi J., Zhang X., Giangrande P.H., McNamara II J.O., Nimjee S.M., Sarraf-Yazdi S., Sullenger B.A., Clary B.M. (2005). Targeted inhibition of αvβ3 integrin with an RNA aptamer impairs endothelial cell growth and survival. Biochem. Biophys. Res. Commun..

[46] Lerner N., Avissar S., Beit-Yannai E. (2017). Extracellular vesicles mediate signaling between the aqueous humor producing and draining cells in the ocular system. PLoS ONE.

[47] Rout S.K., Priya V., Mehata A.K., Muthu M.S. (2022). Abciximab coated albumin nanoparticles of rutin for improved and targeted antithrombotic effect. J. Drug Deliv. Sci. Technol..

[48] Wang X., Gu X., Wang H., Sun Y., Wu H., Mao S. (2017). Synthesis, characterization and liver targeting evaluation of self-assembled hyaluronic acid nanoparticles functionalized with glycyrrhetinic acid. Eur. J. Pharm. Sci..

[49] Aimaletdinov A.M., Gomzikova M.O. (2022). Tracking of extracellular vesicles’ biodistribution: New methods and approaches. Int. J. Mol. Sci..

