# Supplementary material for: Development of 5-Fluorouracil/pH-Responsive Adjuvant-Embedded Extracellular Vesicles for Targeting αvβ3 Integrin Receptors in Tumors
Source: Pharmaceutics. 2024 Apr 29;16(5):599. doi: 10.3390/pharmaceutics16050599 (PMC11125367; doi:10.3390/pharmaceutics16050599)
Supplement: Supplementary file 1 [file pharmaceutics-16-00599-s001.zip › pharmaceutics-2945134-supplementary.pdf]

Supplementary Information

Development of 5-Fluorouracil/pH-Responsive Adjuvant-Embedded Extracellular Vesicles for Targeting $\alpha_v\beta_3$ Integrin Receptors in Tumors

Jiseung Kim [1], Eunsol Lee [1] and Eun Seong Lee [2,*]

[1] Department of Biotechnology, The Catholic University of Korea, 43 Jibong-ro, Bucheon-si 1462, Gyeonggi-do, Republic of Korea; wltmd98@naver.com (J.K.); eunsollee13@gmail.com (E.L.)
[2] Department of Biomedical-Chemical Engineering, The Catholic University of Korea, 43 Jibong-ro, Bucheon-si 1462, Gyeonggi-do, Republic of Korea
* Correspondence: eslee@catholic.ac.kr; Tel.: +82-2-2164-4921

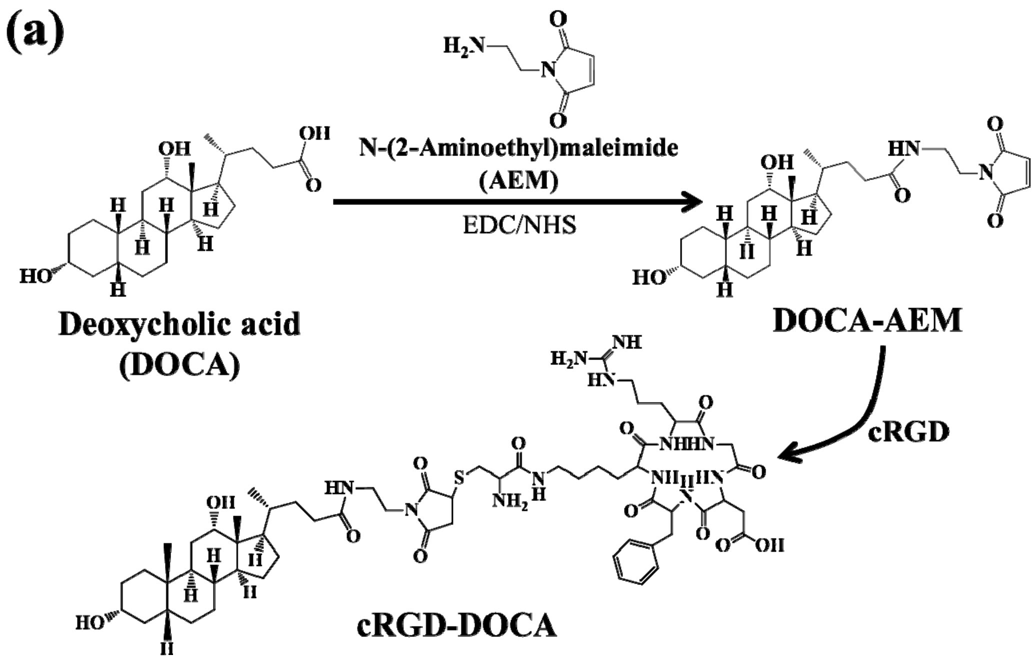

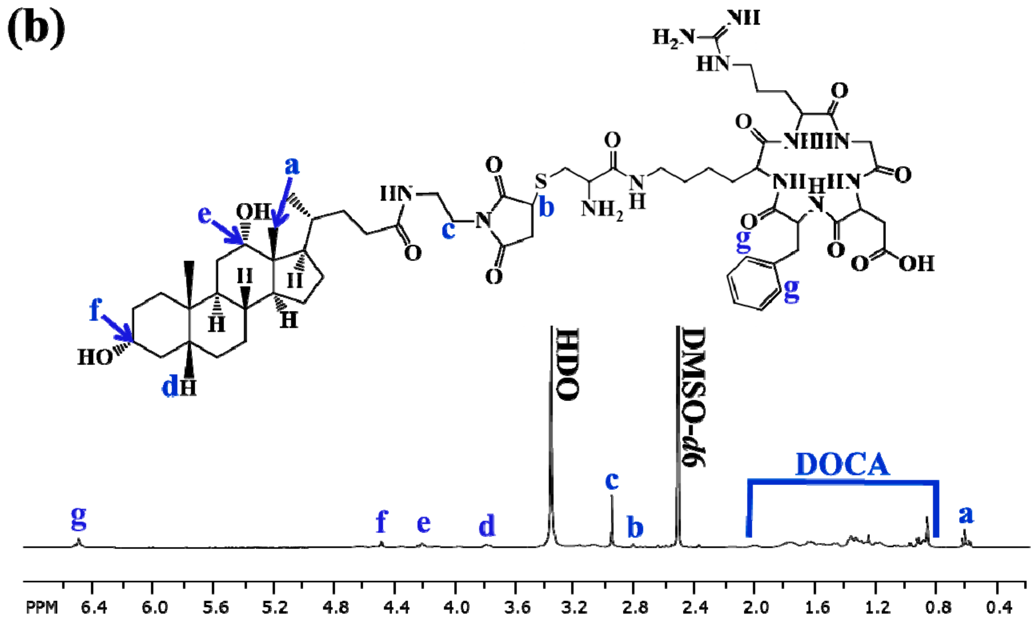

Figure S1. (a) Chemical synthesis scheme of cRGD-DOCA. (b) ^{1}H-NMR peaks of cRGD-DOCA.

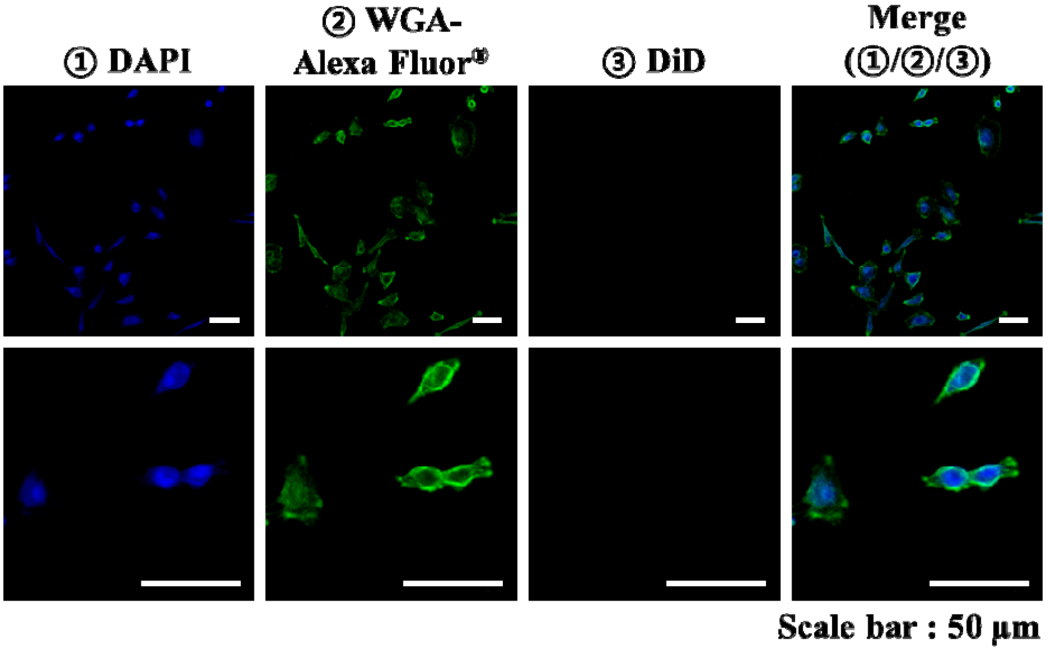

Figure S2. Confocal microscope images of B16BL6 cells treated with (5-FU/DEAP-DOCA/cRGD-DOCA)@EVs (without DiD) for 4 h at 37 °C.
